# Polymorphic Vessels–Think About Seborrheic Keratosis

**DOI:** 10.5826/dpc.1004a90

**Published:** 2020-10-26

**Authors:** Teresa Deinlein, Elena Eber, Regina Fink-Puches, Rainer Hofmann-Wellenhof

**Affiliations:** 1 Department of Dermatology and Venereology, Medical University of Graz, Austria

**Keywords:** seborrheic keratosis, melanoma, dermoscopy, polymorphic vessels

## Case Presentation

**Figure 1:**
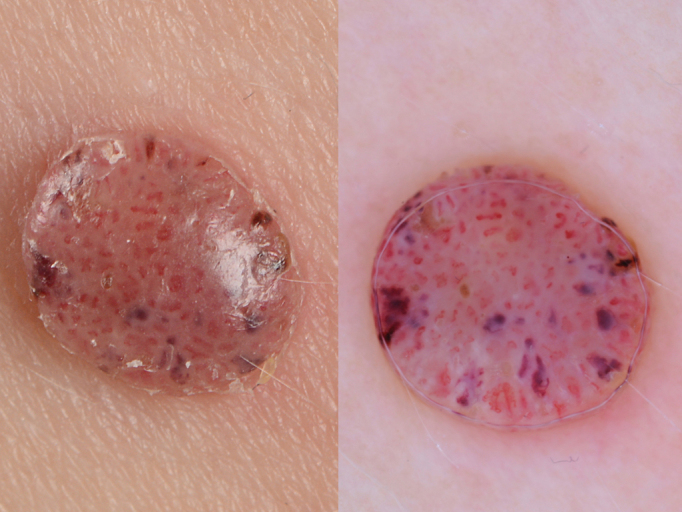
(A) Clinical image showing a roundish, well-demarcated red nodule with a maximum diameter of 8 mm. Additionally, a shiny surface, some peripheral scales, and different types of vessels are observable. (B) On dermoscopy, a polymorphic vascular pattern composed of linear-irregular, glomerular, and hairpin vessels is evident. Moreover, blue-reddish lacunae and hemorrhagic crusts are seen.

A 57-year old man with known multiple atypical nevi presented for his regular yearly follow-up visit. He had no history of personal or familial melanoma. During the visit, we observed a roundish, well-demarcated, nodular lesion with a diameter of 8 mm on his right flank. A shiny surface, some scales at the periphery and clinically visible vessels were observed. The lesion was firm on palpation. On dermoscopy, the lesion presented a polymorphic vascular pattern (linear- irregular, glomerular, and hairpin vessels), blue-reddish lacunae randomly distributed over the lesion as well as some hemorrhagic crusts (Figure 1, A and B).

The nodule was excised, and histopathology showed an irritated seborrheic keratosis with reactive atypia.

## Teaching Point

Seborrheic keratoses, especially irritated lesions, present in a huge morphological variety clinically and dermoscopically. These lesions can exhibit features suggestive of amelanotic melanoma, Merkel cell carcinoma (eg, polymorphic vessels), or basal cell carcinoma and require histological examination [1,2].
